# Response and resilience of anammox consortia to nutrient starvation

**DOI:** 10.1186/s40168-021-01212-9

**Published:** 2022-02-01

**Authors:** Dou Wang, Yulin Wang, Lei Liu, Yiqiang Chen, Chunxiao Wang, Yu-You Li, Tong Zhang

**Affiliations:** 1 Environmental Microbiome Engineering and Biotechnology Laboratory, The University of Hong Kong, Hong Kong SAR, China; 2 Department of Civil and Environmental Engineering, Graduate School of Engineering, Tohoku University, 6-6-06 Aoba, Aramaki, Aoba-ku, Sendai 980-8579, Japan; 3 School of Environment and Energy, Peking University Shenzhen Graduate School, Shenzhen, China; 4 Shenzhen Bay Laboratory, Shenzhen, China

**Keywords:** Anammox consortia, Nutrient starvation, Response, Resilience, Recovery, Transcriptional pattern

## Abstract

**Background:**

It is of critical importance to understand how anammox consortia respond to disturbance events and fluctuations in the wastewater treatment reactors. Although the responses of anammox consortia to operational parameters (e.g., temperature, dissolved oxygen, nutrient concentrations) have frequently been reported in previous studies, less is known about their responses and resilience when they suffer from nutrient interruption.

**Results:**

Here, we investigated the anammox community states and transcriptional patterns before and after a short-term nutrient starvation (3 days) to determine how anammox consortia respond to and recover from such stress. The results demonstrated that the remarkable changes in transcriptional patterns, rather than the community compositions were associated with the nutritional stress. The divergent expression of genes involved in anammox reactions, especially the hydrazine synthase complex (HZS), and nutrient transportation might function as part of a starvation response mechanism in anammox bacteria. In addition, effective energy conservation and substrate supply strategies (ATP accumulation, upregulated amino acid biosynthesis, and enhanced protein degradation) and synergistic interactions between anammox bacteria and heterotrophs might benefit their survival during starvation and the ensuing recovery of the anammox process. Compared with abundant heterotrophs in the anammox system, the overall transcription pattern of the core autotrophic producers (i.e., anammox bacteria) was highly resilient and quickly returned to its pre-starvation state, further contributing to the prompt recovery when the feeding was resumed.

**Conclusions:**

These findings provide important insights into nutritional stress-induced changes in transcriptional activities in the anammox consortia and would be beneficial for the understanding of the capacity of anammox consortia in response to stress and process stability in the engineered ecosystems.

**Video Abstract**

**Supplementary Information:**

The online version contains supplementary material available at 10.1186/s40168-021-01212-9.

## Background

The anaerobic ammonium oxidation (anammox) process is one of the most energy-efficient biotechnologies for nitrogen removal and has therefore been widely implemented in wastewater treatment plants [1]. This process is mediated by a deeply branching group of chemolithoautotrophic bacteria affiliated with the phylum of *Planctomycetes* that are capable of oxidizing ammonium coupled to the reduction of nitrite and the formation of dinitrogen gas in the absence of oxygen [2, 3]. In municipal wastewater treatment plants, the development of stable anammox process is impeded by unpredictable process instabilities due to complex constituents of wastewater and fluctuations in environmental conditions [4–6]. When these unfavorable influencing factors are coupled, deteriorative anammox performance will be observed, and the metabolic activity recovery of anammox consortia from an inhibition event can take up to 6 months [7, 8]. In addition, due to the specific physiological characteristics (e.g., slow growth rates, low cell yields, and high sensitivity to changing environmental conditions) of anammox bacteria, they tend to form complex microbial communities with many potential synergistic and antagonistic interactions during the actual application process [5, 9–12]. Moreover, microbial synergistic interactions have been verified to be central to the stability, maintenance, and functional diversity of microbial communities [13–16]. Therefore, the interpretation of community responses and resilience to external disturbances and microbial community interactions is necessary for the successful full-scale implementation of anammox processes [17, 18].

Numerous studies have elucidated the effects of operational factors (e.g., temperature and dissolved oxygen (DO)) on anammox performance and the microbial community based on 16S rRNA gene or metagenomic analysis [19–22]. Although the microbial composition and metabolic potential can be demonstrated by metagenomic data, the changes in metabolic activities revealed by metatranscriptomic data are also of critical importance to explore how microbes respond to short-term disturbance when the community structure does not change significantly [23]. Understanding the transcriptional responses of anammox consortia during disturbances as well as the synergetic interactions between microorganisms is crucial for understanding the capacity of anammox consortia in response to stress and system stability. The integrated strategy has been used to investigate the stress (e.g., DO, temperature, or nutrient limitation) responses of anammox bacteria at various biological levels and the subsequent recovery process [24–27]. However, few studies have revealed the effect of starvation caused by short-term nutrient deprivation (e.g., caused by system failures) on the anammox system, especially using multi-omics strategies.

In this study, we aimed to explore the responses and resilience of anammox consortia to a short-term nutrient starvation. Metagenomics and metatranscriptomics sequencing were integrated together to investigate the transcriptional response of the core metabolic genes involved in the anammox process, individual organisms and microbial consortia, and the ensuing recovery processes. One lab-scale upflow anaerobic sludge blanket (UASB) anammox reactor that had been operated for almost 2 years suffered a short-term nutrient suspension for 3 days. Four independent granular sludge samples covering the nutrient suspension event, recovery stage, and stable states were collected from this reactor. Our findings illustrated that the anammox consortia exhibited divergent transcriptional responses and resilience to the starvation condition and provided new insights into nutritional stress-induced changes in transcriptional activities of the anammox community. In addition, this study is of great significance to the in-depth understanding of the assembly and interactions of microbial communities in wastewater treatment reactors and can provide guidance for strategies for the recovery of the treatment process and the establishment of appropriate control programs.

## Methods

### Bioreactor operation and sampling

**Fig. 1:**
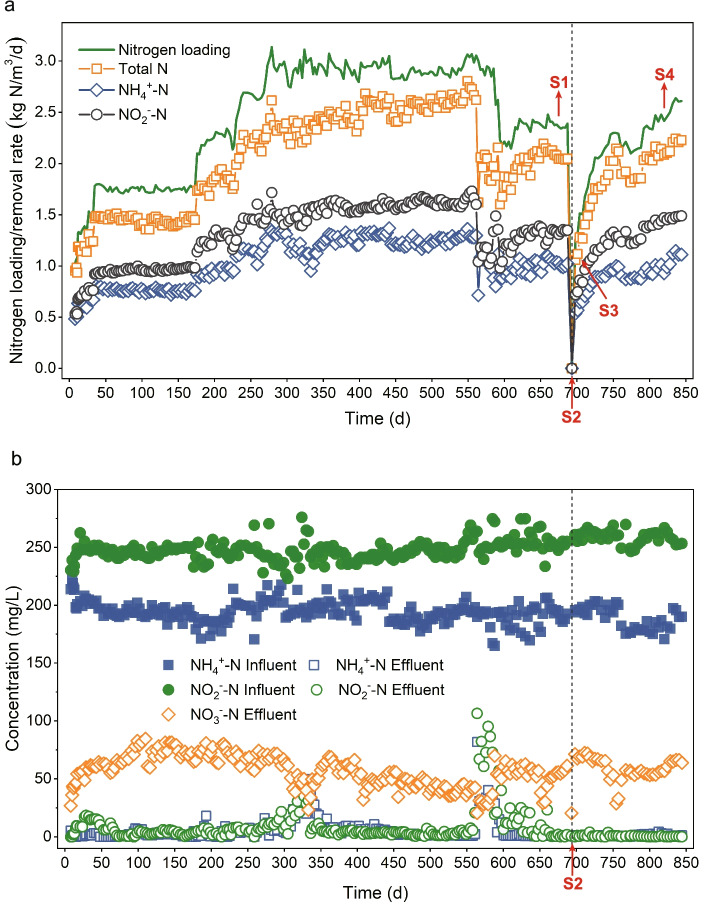
UASB reactor performance. **a** Nitrogen loading rate (green line) and removal rate during the long-term operation. S1–S4, indicated in red, represent the samples collected on Day 680, Day 693, Day 702, and Day 824, respectively. S2 indicates the time point at which the influent was interrupted. **b** Nitrogen concentrations in the influent and the effluent

One lab-scale UASB reactor (anammox bioreactor) with a working volume of 1.5 L was initially inoculated with anammox biomass in December 2017 and has been in operation for almost 2 years before this study. Synthetic wastewater containing ammonium bicarbonate and sodium nitrite was used as the nitrogen source to sustain the growth and reproduction of anammox bacteria. Mineral salt and trace elements were added to provide elements for the microbial growth. The detailed composition of the synthetic wastewater is described in the Supplementary Information. To generate the starvation condition, the influent was interrupted from Day 690 to Day 692, and the influent was then resupplied on Day 693. Samples from four time points (Day 680, Day 693, Day 702, and Day 824) (Fig. 1a) were selected to investigate microbial community dynamics and transcriptional activities before and after influent suspension. Granular sludge samples (granular size: 2.0–3.0 mm, Fig. S1) were collected from the same sampling point in the UASB reactor at each specific time point, and these samples then were frozen in liquid nitrogen and stored at − 80 °C for subsequent DNA and RNA extraction and sequencing. The water parameters of the four sampled time points are listed in Table S1.

### Nucleic acid extraction and sequencing

Total genomic DNA and RNA were extracted from granular sludge using the FastDNA® Spin Kit for Soil (MP Biomedicals, France) and the RNeasy® PowerSoil® Total RNA Kit (QIAGEN, Germany), respectively, according to the manufacturers’ protocols. DNA and RNA were quality-checked by using NanoDrop (Thermo Fisher Scientific, USA). For metatranscriptomic sequencing, rRNA was removed using the Ribo-Zero rRNA removal kit (Illumina, USA), and the remaining mRNA was used to synthesize cDNA by using mRNA template for subsequent sequencing. DNA and cDNA samples from these four time points were sequenced on the Illumina HiSeq PE150 platform to generate 150 bp paired-end reads with 350 bp insert size, and approximately 10 Gb sequencing data for each sample were generated by Novogene (Beijing, China).

### Metagenomic assembly and binning

The paired-end reads from four time points were co-assembled using the MetaWRAP-assembly [28] module by calling the metaSPAdes (v3.13.0) [29] with the default settings. Unsupervised binning was conducted using MetaBAT2 (v2.12.1) [30] and MaxBin2 (v2.2.6) [31], and two bin sets were obtained in this step. The bin_refinement module was employed to consolidate the two bin sets into a comprehensive bin set. CheckM (v1.1.2) [32] was used to estimate the completeness and contamination of the metagenome-assembled genomes (MAGs), and only those MAGs with > 50% completeness and < 10% contamination were selected for the subsequent analyses. The relative abundance of the recovered MAGs was calculated using CoverM (v0.4.0) (https://github.com/wwood/CoverM) with the default settings, where the paired-end reads were mapped directly to the MAGs recovered from four time points. All MAGs recovered in this study have been deposited in the NCBI database under BioProject accession number PRJNA715587, and the genome accession numbers are listed in Table S2.

### Phylogenetic analysis and MAGs annotation

The taxonomic classification of the recovered MAGs and the construction of the corresponding genome tree were implemented by using GTDB-Tk (v1.2.0, reference data version r89) with a concatenated set of 120 bacterial-specific conserved marker genes [33]. The genes and metabolic pathways of the newly recovered MAGs were annotated using the Kyoto Encyclopedia of Genes and Genome (KEGG) GhostKOALA [34]. For the core genes in the anammox reaction that could not be annotated by KEGG, open reading frames (ORFs) predicted from the anammox draft genome were aligned to the reference *Candidatus* Kuenenia stuttgartiensis genome for annotation.

### Peptidase and transporter identification

The peptidases within the recovered MAGs were identified by searching against the MEROPS database (release 12.1) [35], and then the genes that were more than 50% similarity to the reference genes in the database were then extracted for further analysis. The subcellular locations of the peptidases were predicted with PSORTb [36]. Transporters encoded by the recovered MAGs were annotated against TransportDB 2.0 using the automated annotation pipeline TransAAP [37].

### Metatranscriptomic analysis

SortMeRNA (v4.0.0) [38] was applied to remove non-coding RNA sequences in the metatranscriptomic data based on the multiple rRNA databases for bacterial, archaeal, and eukaryotic sequences. Prodigal (v2.6.3) [39] was used for ORF prediction of co-assembled contigs from the metagenomic data and the predicted protein sequences were annotated using KEGG GhostKOALA [34]. For the investigation of gene expression at different time points, transcripts per million (TPM) values were employed in this study, and RSEM [40] was employed for TPM value calculation by mapping non-rRNA reads to all predicted ORFs. The gene expression values of the recovered MAGs were calculated based on the proportion of summed ORF TPM values in a given MAG to the summed TPM values of all predicted ORFs of the co-assembled metagenome. Relative gene expression was calculated by relativizing the median TPM values across all ORFs within the MAG [15]. GFOLD (v1.1.4) [41] was employed to identify differentially expressed genes (DEGs) across different time points, which was especially useful for comparing samples without biological replicates. To minimize the potential noise from marginally responsive genes, the DEG filtering cutoff was set at 0.01 (−sc 0.01), and a |GFOLD value| > 1 was also required [42].

Pathway expression was calculated based on the average expression values of the steps within a pathway. The expression of a step was calculated following these criteria: (a) if a step included an enzyme complex, the average expression of each subunit was employed; and (b) if a step could be catalyzed by more than one enzyme or multiple copies of an enzyme, then the summed expression was adopted [43].

## Results

### Reactor performance before and after starvation

The performance of the reactor was steadily improved after inoculation, and nitrogen loading was continually increased and finally achieved a robust nitrogen removal at approximately 200 days, with a removal rate of 1.8 kg N/m^3^/day (Fig. 1a and b) and removal efficiency of ~ 80% (Fig. S2), although there were some fluctuations during the long-term operation. To investigate the starvation response of the anammox reactor, the influent was interrupted for 3 days from Day 690 to Day 692. Subsequently, to reactivate the reactor, the influent was resupplied on Day 693, and nitrogen loading was gradually increased to avoid possible substrate inhibition. A high-quality effluent (< 2 mg/L NH_4_^+^-N) and a high removal efficiency (> 84%) were observed after the influent was resupplied, and the nitrogen removal rate increased with the loading rate without significant stagnation, indicating that the anammox system was resilient and robust to dealing with this short-term nutritional stress.

### Microbial community dynamics and transcriptomic expression

**Fig. 2:**
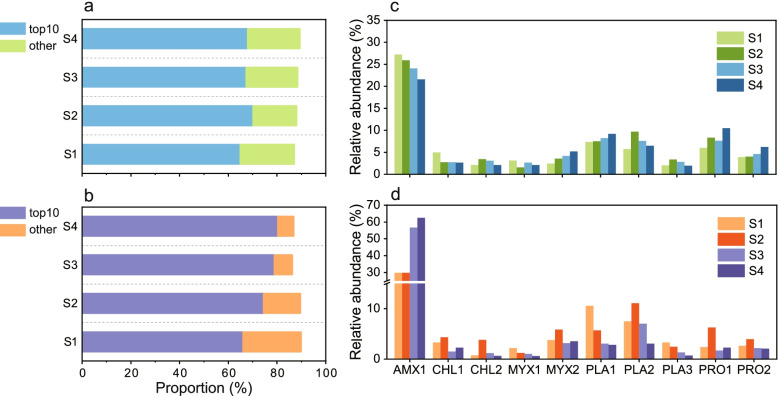
Relative abundance and gene expression profiles of recovered MAGs. **a** The proportion of the top 10 most abundant MAGs and other recovered MAGs in the whole community calculated based on the relative abundance of individual MAGs. **b** The proportion of gene expression of the top 10 most abundant MAGs and other recovered MAGs in the whole community calculated based on the expression of individual MAGs. **c** The relative abundance (normalized by the genome size) of the top 10 most abundant MAGs at different time points. **d** Gene expression of the top 10 most abundant MAGs at different time points. See Methods section for calculation details

A total of 88 bacterial MAGs were retrieved from the metagenome. These retrieved MAGs accounted for nearly 87–89% (Fig. 2a) and 83–89% (Fig. 2b) of the total filtered metagenomic reads and transcripts obtained from the four sampled time points, demonstrating that the recovered MAGs represented the majority of the populations in the anammox system. These 88 MAGs were mainly affiliated with the phyla of *Proteobacteria* (24), *Planctomycetes* (9), *Chloroflexi* (18), and *Acidobacteria* (9) (Table S2 and Fig. S3). As shown in Fig. 2c, the anammox bacterium (AMX1) affiliated with *Candidatus* Kuenenia stuttgartiensis dominated the microbial community across these four studied biomass samples, although it showed a slight decrease in abundance after influent suspension (from 27.2 to 21.6%). PLA1, PLA2, PRO1, and PRO2 were also predominant populations in the microbial community at all time points and showed a minor increase in abundance relative to the pre-starvation stage. To assess the variation in the microbial composition, the Bray-Curtis distances of four metagenomes were calculated, and the relatively lower Bray-Curtis distances of the microbial composition between S1 and the other three time points (S2: 0.12, S3: 0.11, and S4: 0.21) indicated that the microbial community composition was resistant to the short-term starvation, although a slight variation in the relative abundance was induced. Moreover, the gene expression of individual MAGs was investigated to assess the response of these highly abundant microbes (Fig. 2d). The transcriptional expression of AMX1, which was in line with its abundance, dominated the microbial community, and its expression was highly upregulated with the increase in nitrogen loading after the feeding was resumed. The top 9 heterotrophs accounted for 18–44% of the total transcripts at different time points. Intriguingly, several genomes showed higher activity at S2, including CHL1, CHL2, MYX2, PLA2, PRO1, and PRO2, after which they presented a noticeable decrease in transcriptional activity when nutrients were resupplied, although some increases in relative abundance were observed.

### Transcriptional response of core genes involved in the anammox process

**Fig. 3:**
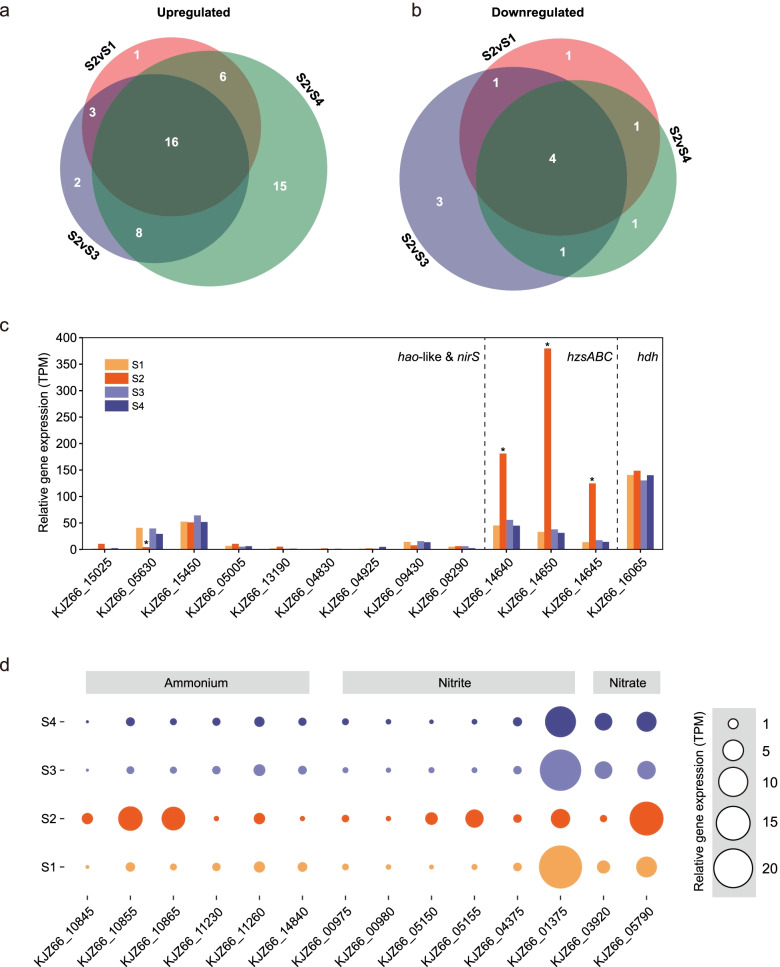
Expression profiles of genes involved in the anammox process. **a** Venn diagram indicating the genes encoded by AMX1 were significantly upregulated (GFOLD value > 1) at S2 compared with the other three time points. **b** Venn diagram indicating the genes encoded by AMX1 were significantly downregulated (GFOLD value < -1) at S2 compared with the other three time points. **c** The relative gene expression of key genes possibly involved in anammox reactions. Asterisks indicate the genes that were differentially expressed between S2 and the other three time points (except *hzsA* S2 vs. S3). **d** The relative gene expression (indicated by bubble diameter) of ammonium transporters, nitrite transporters and nitrate transporters at different time points

As the central biocatalysts in this microbial ecosystem, the transcriptional response of anammox bacteria to the nutrient starvation is of great interest. DEGs of AMX1 were identified by comparing the transcript profiles of the four time points. When S2 was compared with the other three time points, 26, 29, and 45 genes were significantly upregulated (GFOLD > 1) (Fig. 3a), respectively, including 16 genes that were consistently upregulated at S2. In addition, 7, 9, and 7 genes were downregulated (GFOLD < -1) (Fig. 3b), including 4 genes that were consistently downregulated compared with the other three time points. Among these DEGs, some indispensable genes performing anammox reactions were included, such as the *hao*-like, hydrazine synthase complex (*hzsABC*), and hydrazine dehydrogenase (*hdh*) genes.

Eight *hao*-like genes and one nitrite reductase (*nirS*) gene were identified in AMX1 (Fig. 3c, Table S3). The two highly expressed *hao*-like genes (KJZ66_05630 and KJZ66_15450) and the low expression of the *nirS* gene (KJZ_08290) in both the steady states and the recovery state suggested that these *hao*-like genes might encode physiological nitrite reductases that reduce nitrite to nitric oxide [44]. In this study, KJZ66_05630 and KJZ66_09430 were found to be identical to kustc0458 and kuste4574, corroborating their potential nitrite reduction ability [45]. Interestingly, significantly downregulated expression of KJZ66_05630 was observed when the influent was suspended. The concentration of nitrite at the sampling point was only 0.72 mg/L when the influent was suspended, which might explain the downregulated expression of KJZ66_05630. KJZ66_15450, which is identical to Kustc1061 in *Candidatus* Kuenenia stuttgartiensis genome, was the most active *hao*-like gene in the recovered AMX1 genome [45]. Following the reduction of nitrite to nitric oxide, the conversion of nitric oxide and ammonium to hydrazine is carried out by the *hzs* complex. Notably, genes encoding *hzsABC* were significantly upregulated (Fig. 3c, Table S3) in response to nutrient starvation at stage S2. The gene expression of KJZ66_14650 (*hzsB*) at S2 increased by approximately 10- to 11-fold compared with the other time points. However, the expression of genes encoding hydrazine dehydrogenase, catalyzing hydrazine oxidation, remained stable across all four sampling points (Fig. 3c).

### Transcriptional activities of nutrient transporters in anammox bacteria

All reported anammox genomes harbor multiple copies of nutrient transporters to facilitate the transport of major substrates [26]. Six genes encoding ammonium transporters were identified in AMX1 (Fig. 3d, Table S2), and all of them belong to prokaryotic Rh members of the AmtB/Mep/Rh transporter family [26]. Three of these ammonia transporters (KJZ66_10845, KJZ66_10855, and KJZ66_10865) were identified on a single contig, which were remarkably upregulated at S2, especially for KJZ66_10855 and KJZ66_10865. In contrast, the genes encoding the other three ammonia transporters did not show divergent transcriptional responses to the short-term nutrient suspension.

Regarding nitrite transportation, AMX1 has six genes encoding nitrite transporters from the Format/Nitrite Transporter (FNT) family (Fig. 3d, Table S3), FocA/NirC. Interestingly, the genes encoding nitrite transporters showed divergent transcriptional responses to the nutrient starvation. The most highly expressed nitrite transporter (KJZ66_01375) in the steady states (S1 and S4) and during the recovery process (S3) from nutrient starvation showed approximately 2.8- to 5.9-fold decreases within the period of influent suspension (S2). In contrast, the other two nitrite transporters (KJZ66_05150 and KJZ66_05155) were significantly upregulated in response to nutrient starvation despite the low transcriptional levels observed under normal operation. Other transporters, KJZ66_00975, KJZ66_00980, and KJZ66_04375, showed extremely low expression in all operation stages. Two nitrate transporters were identified in AMX1, both belonging to the NarK2-type, among which KJZ66_05790 was stably expressed under the normal nutrient supply state but showed significant upregulation at stage S2.

### Divergent transcriptional response of high-abundance microbes

**Fig. 4:**
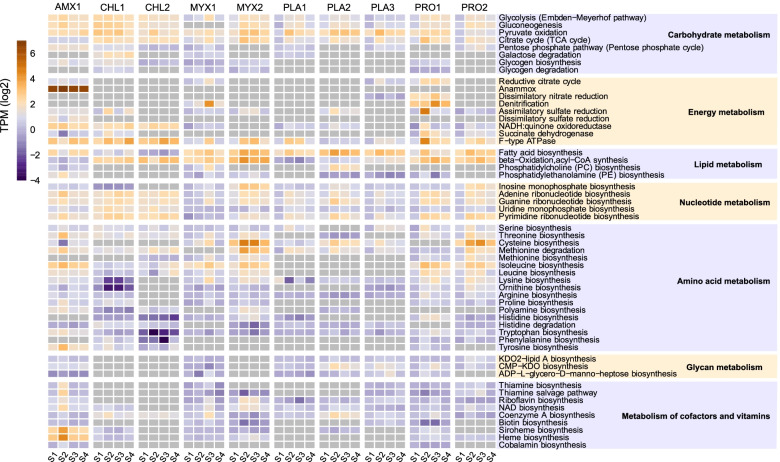
Transcriptional activity of KEGG modules across the top 10 most abundant MAGs. The relative gene expression of KEGG modules were calculated; see the Methods section for calculation details. Gray box indicates module absence

In this study, how the anammox consortia responded to nutrient starvation was also explored by examining the changes in the transcriptional activities of both the whole community and individual members based on different KEGG metabolism pathways (Fig. S4). Consistent with our expectations, the community-wide transcriptional activity related to energy metabolism decreased at S2, which was in line with the nutrient starvation state at this time point. Moreover, the metabolic activities related to nucleotides, amino acids, cofactors, and vitamins were lower than those in S1, S3, and S4, while the transcriptional activities related to translation, protein folding, sorting and degradation were remarkably elevated at S2. To determine how individual community members contributed to the observed community-wide transcriptional pattern, KEGG modules were employed to explore the transcriptional patterns of the top 10 most abundant community members in different experimental phases (Fig. 4).

When anammox consortia were subjected to starvation stress, upregulated expression of glycolysis/gluconeogenesis, pyruvate oxidation, and TCA cycle pathways could be observed in most of the abundant organisms (except that the pyruvate oxidation pathway was missing in MYX1), illustrating that these microbes tended to generate more intermediates for self-maintenance [46]. Relatively high degradation activity toward glycogen, a storage compound that could help organisms cope with substrate fluctuations, was observed in CHL1 and MYX2 [47]. In PLA3 and PRO1, upregulated expression of the dissimilatory nitrate reduction pathway was found at S3, and a similar trend for denitrification process was observed in MYX1 and PRO1. This profile was in accordance with the expression of key genes involved in the denitrification process (Fig. S5). Interestingly, all abundant heterotrophs except for PLA2 showed unregulated ATP biosynthesis at S2 to maintain basic cellular reactions [46].

**Fig. 5:**
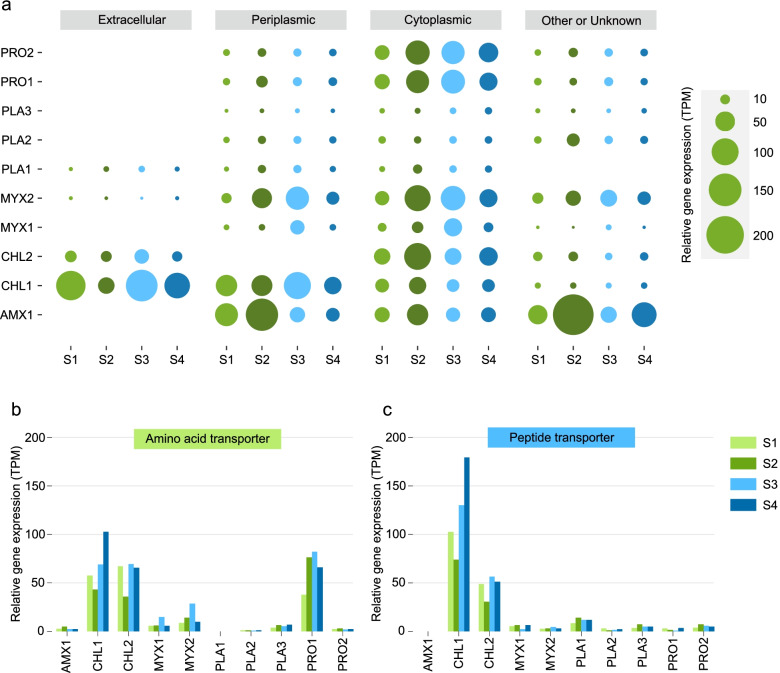
Transcriptional activity of predicted peptidases, amino acid, and peptide transporters among the top 10 most abundant MAGs at different time points. **a** The relative gene expression of peptidases across the MAGs. The peptidases in these recovered MAGs were identified by searching against the MEROPS database, and the locations of peptidases were predicted by PSORTb. **b** The relative gene expression of amino acid transporters predicted across the MAGs. **c** The relative gene expression of peptide transporters across the MAGs. Transporters encoded by the recovered MAGs were annotated against TransportDB 2.0 using the automated annotation pipeline TransAAP

Amino acids and vitamins have been characterized as important metabolite intermediates that shape microbial community assembly [13, 15, 48]. In the anammox consortia, the majority of abundant organisms (e.g., AMX1, CHL1, MYX1, MYX2, PRO1, and PRO2) expressed genes for synthesis of most amino acids, most of which were highly expressed at the stages S2 and S3 in particular. In contrast to these microbes, PLA1, PLA2, and PLA3 were missing pathways for the synthesis of numerous crucial amino acids. Furthermore, for the metabolic profile of AMX1, genes related to the synthesis of amino acids with great metabolic cost (e.g., isoleucine and tyrosine) were highly expressed (especially at S2); however, most of the heterotrophs showed lower activity of these pathways or were missing them, indicating that these heterotrophs might obtain these amino acids from AMX1. In addition to de novo synthesis, protein degradation may be another source of amino acids. Notably, peptidase transcripts in AMX1 increased by 3-fold at S2, suggesting that anammox might degrade more proteins to increase the availability of amino acids required for the synthesis of new proteins [49]. Additionally, many of the abundant organisms (e.g., CHL1, CHL2, MYX2, and PLA1) might be highly active extracellular protein degraders (Fig. 5a, Table S4). In particular, CHL1 and CHL2 showed high expression of genes encoding amino acid and peptide transporters despite their downregulation at S2 (Fig. 5b and c, Table S5). In contrast to the response of peptidase genes, the transcriptional activity of diverse glycoside hydrolase genes involved in the hydrolysis of carbohydrate bonds was stable at all time regimes (Fig. S6). Furthermore, the transcriptional profiles demonstrated that some heterotrophs were able to synthesize cofactors and vitamins, but the expression of related genes was not prominent at all the four time points.

### Transcriptional resilience of anammox bacteria and heterotrophs

**Fig. 6:**
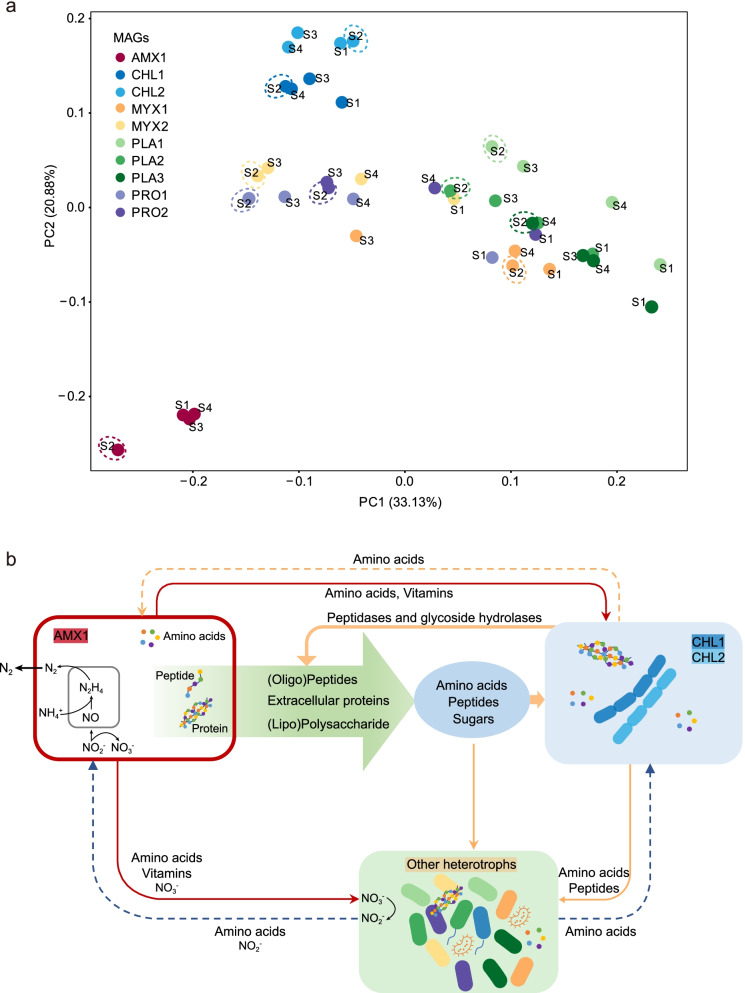
Transcriptional patterns and proposed metabolic interactions of the top 10 most abundant MAGs at different time points. **a** Principal coordinates analysis (PCoA) (based on Bray-Curtis distance) based on the expression profiles of KEGG modules illustrating the transcriptional pattern of each MAG. The dashed line highlights the time point of the influent interruption. **b** The proposed metabolic interactions among the core autotrophic producers (AMX1), secondary producers (CHL1 and CHL2), and other abundant heterotrophs

To evaluate the transcriptional resilience of these abundant microbes to nutrient starvation, principal coordinates analysis (PCoA) was conducted based on the transcriptional profiles of the studied KEGG modules (Fig. 6a). For AMX1, a distinct clustering distance was observed between the starvation and normal nutrient supply conditions, and the transcriptional pattern could quickly return to a similar-to-pre-starvation state once the nutrient became available again, as evidenced by a tight clustering pattern of the time points of S1, S3, and S4 and the low Bray-Curtis distance (0.04) between S1 and S4 (S1 vs. S2: 0.27) (Table S6). However, in contrast to AMX1, the abundant heterotrophs in the studied anammox reactor showed distinct transcriptional resilience. Although relatively tight clustering of CHL1 and CHL2 was observed at the different points compared with the other heterotrophs, the transcriptional patterns were also distinct between S1 and S4. For other heterotrophs, the discrete clustering patterns of different time points and distinct distances between S1 and S4 implied lower transcriptional resilience. Nevertheless, it is assumed that the transcriptional patterns of these microorganisms at S4 were approaching the transcriptional pattern of the stationary phase.

## Discussion

### High resistance and resilience of the anammox consortia to starvation

By comparing the anammox performance and gene expression profile before and after the starvation, we could gain a comprehensive understanding of the resistance capacity and response mechanisms of anammox consortia under nutrient starvation. Short-term starvation did not have an irreversible effect on anammox performance, and no substrate inhibition was observed after the influent was resupplied, indicating that anammox bacteria could rapidly metabolize the substrate and recover promptly from short-term nutrient starvation. Consistent with our expectations, no apparent community composition shifts were observed, except for some variations in the relative abundance. Intriguingly, the different trends (from S1 to S4) in the relative abundance of some heterotrophs (increases in MYX2, PLA1, PRO1 and PRO2, and decreases in CHL1 and MYX1) suggest the divergent abilities to compete for resources during the starvation and the recovery process [50], and the decreased relative abundance of anammox bacteria implies that their cell detritus may be a resource for these heterotrophs [15]. Despite the decrease in abundance, anammox bacteria maintained a high transcriptional activity at stages S3 and S4, corresponding to the recovered performance of the anammox process.

### Active response of anammox bacteria to nutrient starvation

The expression pattern showed that AMX1 maintained a high transcriptional level at S2, which was consistent with the energetic transcriptional responses of some key genes involved in the anammox process. Elevated transcriptional activity of the *hzs* comple*x* and stable high expression of the *hdh* gene during the influent suspension period probably represents an adaptation mechanism to the starvation condition. A similar upregulation of the *hzs* genes was also observed when the anammox bacteria were subjected to ammonium or nitrite limitation conditions [26, 51]. In particular, divergent responses of the *hzs* complex and *hdh* gene to other shocks or disturbances have been discovered. In contrast to our observations in this study, decreased transcriptional activities of the *hzs* complex and upregulation or downregulation of *hdh* gene expression were observed when the anammox bacteria were subjected to a lower temperature regime (20 °C to 14 °C) or DO exposure [24, 25]. These effective regulation strategies are able to alleviate the negative impacts of stress conditions on the anammox bacteria.

In addition to the core genes involved in anammox reactions, the genes encoding nutrient transporters responded differently to various nutrient conditions. As alluded to above, some transporter genes (e.g., KJZ66_10855, KJZ66_10865, KJZ66_05155, and KJZ66_05790) were only highly expressed under nutrient starvation condition, indicating that the proteins encoded by these genes may have high affinity to the substrate; a similar highly expressed ammonium and nitrite transport system was also observed in marine *Scalindua* anammox bacteria under substrate limitation [27]. The collaboration of transporter genes with different affinities enables anammox bacteria to maintain their metabolic activity under different nutrient conditions.

Moreover, the high expression of peptidases observed at S2 suggested that anammox bacteria may degrade some less needed proteins to ensure the availability of amino acids for synthesis of the proteins which are more needed for survival under the starvation condition. Taken together, our findings indicated that short-term starvation did not have irreversible negative effects on the anammox consortia, and the high expression of key genes involved in energy metabolism, nutrient transportation, and protein degradation processes implied that these transcriptional responses might play a crucial role in survival under the starvation condition and in the ensuing recovery process. More importantly, the expression profiles of key genes involved in anammox reactions under the starvation condition could be used as biological indicators of system substrate concentrations in the large-scale reactors to guide the establishment of the corresponding substrate supply strategies to achieve a better system operating state.

### Synergistic interactions of anammox consortia to resist nutrient starvation

Synergistic interactions among different populations of the anammox consortia were critical for this ecosystem to maintain stability and resist to the nutrient starvation. As mentioned above, the abundant heterotrophs were active organisms, accounting for 18–44% of the total transcripts. Genome characterization verified that these microorganisms and anammox bacteria shaped the skeleton frame/backbone of a nearly closed N loop (Fig. 6b) and could improve overall nitrogen removal [12]. For denitrification, the high transcriptional activity of genes responsible for reducing nitrate to nitrite in some heterotrophs (e.g., MYX1, PLA2, and PRO1) (Fig. S5) may be important for anammox activity, especially during the period of nutrient depletion. Consistent with this result, a markedly decreased nitrate concentration in liquor (Table S1) was observed at S2, supporting this hypothesis. Associated with this response mechanism, the upregulation of denitrification-related expression in these organisms may be a survival strategy when the consortium is subjected to the starvation condition. The generated nitrite could combine with ammonia derived from protein degradation to drive the anammox reaction, then these denitrification organisms may benefit from the anammox bacteria to complete their own proliferation.

In addition to substrates involved in the central energy metabolism, insights into the exchange of metabolic intermediates between microbial community members under stressful conditions are also of great significance for exploring the survival strategies of different species. For anammox bacteria, in addition to the upregulated expression of the amino acid biosynthesis pathway at S2, the high expression of peptidase genes may result in the generation of more amino acids for the synthesis of new proteins to resist stress and support other heterotrophs under the nutrient starvation condition [49, 52]. Besides, heterotrophs (e.g., CHL1 and CHL2) were capable of degrading extracellular proteins produced by anammox bacteria (Fig. 6b), yielding amino acids available to themselves and other microbial community members [15, 53]. In particular, the high expression of genes related to the transportation of amino acids or peptides was discovered in CHL1, CHL2, and PRO1, allowing them to use these amino acids as a carbon and energy source to synthesize crucial proteins.

More interestingly, the relative gene expression of peptidases in PLA3 was extremely low, while the upregulated gene expression of amino acid and peptide transporters at S2 implied that PLA3 could take advantage of substrates madeavailable from other species to survive. In addition, the higher transcriptional activity related to the cysteine biosynthesis pathway observed in abundant heterotrophs at S2 demonstrated that they may supply this amino acid to anammox bacteria. In summary, the transcriptional response related to the denitrification process, amino acid biosynthesis, peptidase genes, and amino acid and peptide transporters observed in anammox bacteria and other heterotrophs may play an important role in maintaining community activity to survive in a difficult period and lay the foundation for subsequent rapid recovery.

### Distinct transcriptional resilience of anammox bacteria and heterotrophs

According to the transcription profile dissimilarity analysis of different KEGG modules, differences in transcriptional resilience could be observed among anammox bacteria and heterotrophs when they were simultaneously subjected to the nutrient starvation.

The transcriptional pattern of anammox bacteria, which were the core autotrophic primary producers in the studied ecosystem, could rapidly recover to a similar state to the pre-starvation phase after the nutrient resupply [12]. Furthermore, as discussed above, the elevated transcriptional activity of substrate-converting genes (such as the *hzs* complex) and peptidase genes in anammox bacteria implied that they were ready to function as soon as a new pulse of the substrate became available [54].

In heterotrophs such as CHL1 and CHL2, which were the potential secondary producers (extracellular protein degraders) in this ecosystem (Fig. 6b), the relatively high expression of peptidase genes, amino acid, and peptide transporters detected at all time points supports the observation that no obvious shift in the transcriptional pattern (S1 vs. S2) was discovered when the consortia were subjected to the starvation, especially for CHL2. However, the distinct distance between S1 and S4 was observed (Fig. 6a). Combined with the variations in the relative abundance of these organisms, it can be presumed that the competition from other heterotrophs intensified during the recovery process. Similar results have been reported and showed that a decrease in the relative abundance of taxa that were resistant to disturbance may be accompanied by an increase in the relative abundance of some rapidly growing bacteria during the recovery process [50].

Nevertheless, distinct transcriptional patterns were noticed for the other heterotrophs with less resilience (more conspicuous and persistent impacts). Previous studies have demonstrated that heterotrophs realized changes in enzyme activities and ribosome contents via changes in transcription and translation processes related to different substrate concentrations [54]. Therefore, they have to modulate their enzyme patterns to reinstate themselves when a corresponding substrate becomes available. Furthermore, these heterotrophs coexisting in anammox consortia have to rely on anammox bacteria or other active protein degraders (e.g., CHL1 and CHL2) to supply substances for their growth under starvation, which may partially explain the less transcriptional resilience of these species (Fig. 6b). Overall, the transcriptional patterns of these heterotrophic microbes gradually returned to the pre-starvation state, and the functionality of this consortium, assessed based on reactor parameters (e.g., effluent parameters, removal rates, and removal efficiency), remained unchanged. Based on the transcriptional patterns of the anammox consortia and stable anammox performance, it could be inferred that anammox consortia possess relatively high capacity to resist stress and that substrate loading could be gradually increased to the original level after suffering from the nutrient starvation.

## Conclusions

An integrated metagenomics and metatranscriptomics approach was used to explore the response and resilience of anammox consortia to the nutrient starvation. Short-term nutrient starvation did not have an irreversible impact on the system performance. The vigorous nutrient starvation response mechanisms (ATP accumulation, upregulated amino acid biosynthesis, and enhanced protein degradation) and synergistic interactions between the anammox bacteria and other heterotrophs benefited the survival and ensuing recovery process. Short-term nutrient starvation induced divergent transcriptional patterns although there were no remarkable shifts in the microbial community composition, and anammox bacteria showed higher transcriptional resilience than other heterotrophs. These findings regarding the transcriptional response to the starvation will be beneficial to the understanding of the capacity of anammox consortia in response to starvation and process stability in engineered ecosystems. Further studies of long-term nutrient starvation and the corresponding changes in metabolite levels could help better understand the stress responses of anammox consortia.

## Supplementary Information

**Additional file 1: Fig. S1.** The image of the granular sludge collected at the sampling point. **Fig. S2.** Nitrogen removal efficiency during the whole operation. **Fig. S3.** Phylogenetic tree of all recovered draft genomes from the anammox reactor. The tree created based on the concatenated alignment of 120 single-copy marker genes using GTDB-Tk [1]. The generated phylogenetic tree was imported into the Interactive Tree of Life (iTOL) (https://itol.embl.de) for the tree annotation and interpretation [2]. Tree includes MAGs recovered from this study (red and black, red represent the top 10 most abundant organisms) and closely related genomes downloaded from GTDB. Branch with node represents bootstrap support value > 90%. **Fig. S4.** Community-level based metabolism pathway expression at different time points. S1-S4 represent the samples collected on Day 680, Day 693, Day 702, and Day 824, respectively. S2 indicates the time point when the influent was suspended. **Fig. S5.** Genes involved in denitrification process. a, The expression of denitrification genes across the whole community at different time points. b, Presence and relative expression (log2 TPM) of denitrification genes across the top 9 recovered heterotrophic genomes. Gray box indicates gene absence. S1-S4 represent the samples collected on Day 680, Day 693, Day 702, and Day 824, respectively. S2 indicates the time point when the influent was suspended. **Fig. S6.** The relative gene expression of glycoside hydrolases across the recovered MAGs at different time points. The glycoside hydrolases in these recovered MAGs were identified by using HMMER [3] searching against the dbCAN database (V9) [4], and the locations of glycoside hydrolases were predicted by PSORTb [5].

**Additional file 2.**

## Data Availability

All MAGs recovered in this study have been deposited in the NCBI database under BioProject accession number PRJNA715587.

## Abbreviations

DO: Dissolved oxygen
UASB: Upflow anaerobic sludge blanket
MAGs: Metagenome-assembled genomes
ORFs: Open reading frames
TPM: Transcripts per million
DEGs: Differentially expressed genes
*hzs*: gene of hydrazine synthase
*hdh*: gene of hydrazine dehydrogenase
*nirS*: gene of nitrite reductase
PCoA: Principal coordinates analysis
