# Supplementary material for: Response and resilience of anammox consortia to nutrient starvation
Source: Microbiome. 2022 Feb 1;10:23. doi: 10.1186/s40168-021-01212-9 (PMC8805231; doi:10.1186/s40168-021-01212-9)
Supplement: Supplementary file 2 — Additional file 1: Fig. S1. The image of the granular sludge collected at the sampling point. Fig. S2. Nitrogen removal efficiency during the whole operation. Fig. S3. Phylogenetic tree of all recovered draft genomes from the anammox reactor. The tree created based on the concatenated alignment of 120 single-copy marker genes using GTDB-Tk [1]. The generated phylogenetic tree was imported into the Interactive Tree of Life (iTOL) (https://itol.embl.de) for the tree annotation and interpretation [2]. Tree includes MAGs recovered from this study (red and black, red represent the top 10 most abundant organisms) and closely related genomes downloaded from GTDB. Branch with node represents bootstrap support value > 90%. Fig. S4. Community-level based metabolism pathway expression at different time points. S1-S4 represent the samples collected on Day 680, Day 693, Day 702, and Day 824, respectively. S2 indicates the time point when the influent was suspended. Fig. S5. Genes involved in denitrification process. a, The expression of denitrification genes across the whole community at different time points. b, Presence and relative expression (log2 TPM) of denitrification genes across the top 9 recovered heterotrophic genomes. Gray box indicates gene absence. S1-S4 represent the samples collected on Day 680, Day 693, Day 702, and Day 824, respectively. S2 indicates the time point when the influent was suspended. Fig. S6. The relative gene expression of glycoside hydrolases across the recovered MAGs at different time points. The glycoside hydrolases in these recovered MAGs were identified by using HMMER [3] searching against the dbCAN database (V9) [4], and the locations of glycoside hydrolases were predicted by PSORTb [5]. [file 40168_2021_1212_MOESM2_ESM.docx]

**Supplementary Information**

**Response and resilience of anammox consortia to nutrient starvation**

Dou Wang^1^, Yulin Wang^1^, Lei Liu^1^, Yiqiang Chen^1^, Chunxiao Wang^1^, Yu-You Li^2^, Tong Zhang^1,3,4^*

^1^Environmental Microbiome Engineering and Biotechnology Laboratory, The University of Hong Kong, Hong Kong SAR, China.

^2^Department of Civil and Environmental Engineering, Graduate School of Engineering, Tohoku University, 6-6-06 Aoba, Aramaki, Aoba-ku, Sendai 980-8579, Japan.

^3^School of Environment and Energy, Peking University Shenzhen Graduate School, Shenzhen, China.

^4^Shenzhen Bay Laboratory, Shenzhen, China.

E-mail: zhangt@hku.hk; Tel. 852-28578551; Fax 852-25595337.

**Methods**

**Reactor operation**

One lab-scale UASB reactor (anammox bioreactor) was initially inoculated with anammox biomass from Tohoku University (Civil and Environmental Engineering) and has been in operation for almost 2 years before this study. Synthetic wastewater containing NH_4_HCO_3_ and NaNO_2_ is the main nitrogen source. The pH of the influent was controlled at around 7.5-7.8 by NaHCO_3_. And additional mineral medium and trace elements were added to provides elements needed for microbial growth. The composition of the mineral medium was as follows: KCl 0.57 g/L, CaCl_2_·2H_2_O 150 mg/L, 25 mg/L of KH_2_PO_4_, 100 mg/L of MgSO_4_·7H_2_O. The trace elements included Na_2_·EDTA·2H_2_O 19 mg/L, Na_2_WO_4_·2H_2_O 0.664 mg/L, ZnSO_4_·7H_2_O 0.538 mg/L, CoCl_2_·6H_2_O 0.3 mg/L, MnCl_2_·4H_2_O 1.125 mg/L, CuSO_4_·5H_2_O 0.313 mg/L, (NH_4_)_6_Mo_7_O_24_·4H_2_O 0.275 mg/L, NiCl_2_·6H_2_O 0.238 mg/L, Na_2_SeO_4_·10H_2_O 0.123 mg/L, H_3_BO_3_ 0.018 mg/L.


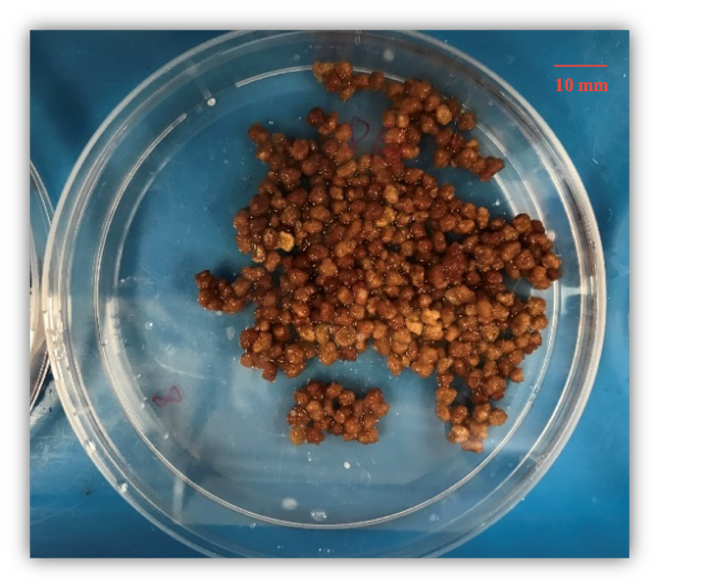


**Fig. S1** The image of the granular sludge collected at the sampling point.





**Fig. S2** Nitrogen removal efficiency during the whole operation


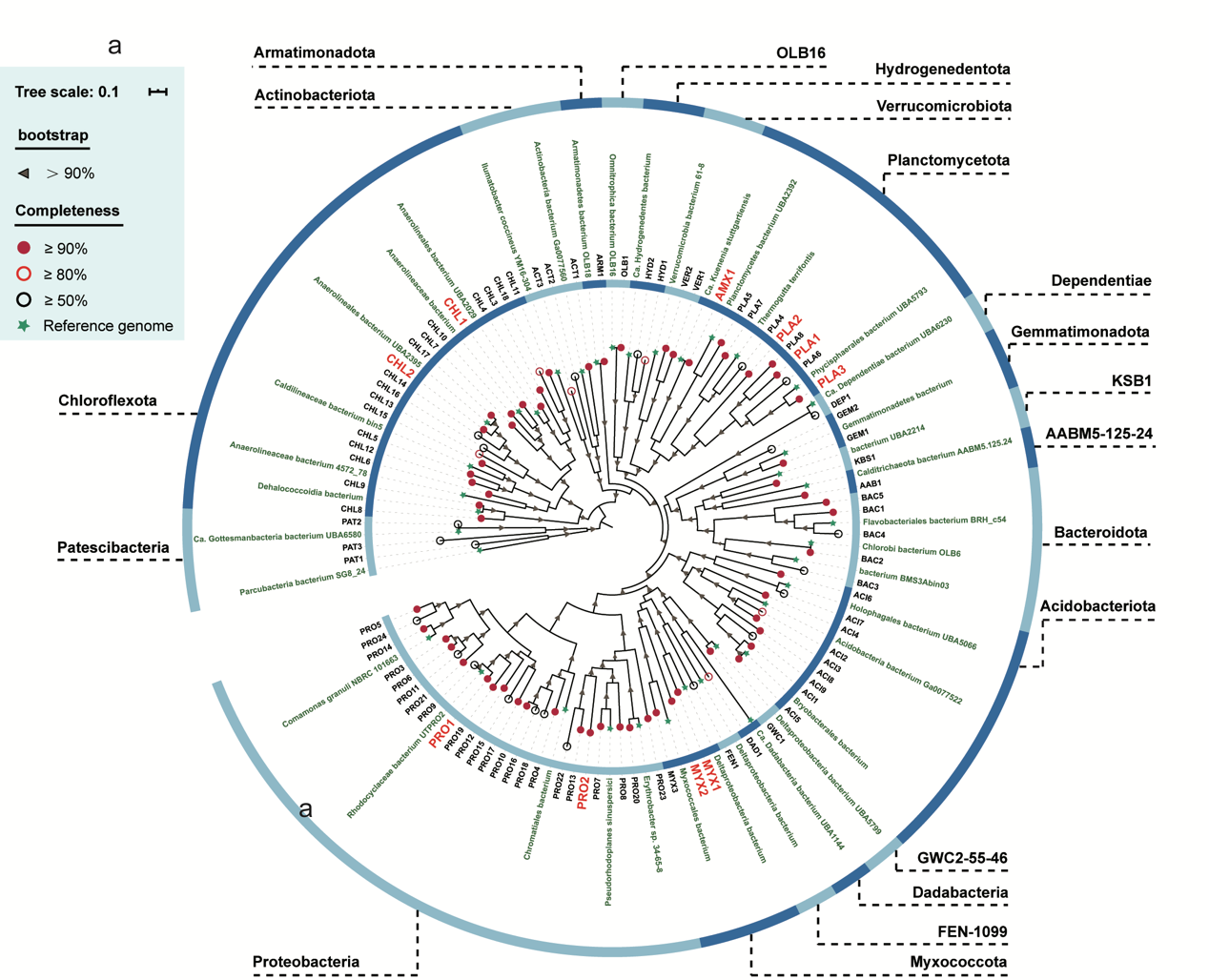


**Fig. S3** Phylogenetic tree of all recovered draft genomes from the anammox reactor. The tree created based on the concatenated alignment of 120 single-copy marker genes using GTDB-Tk [1]. The generated phylogenetic tree was imported into the Interactive Tree of Life (iTOL) ([https://itol.embl.de](https://itol.embl.de/)) for the tree annotation and interpretation [2]. Tree includes MAGs recovered from this study (red and black, red represent the top 10 most abundant organisms) and closely related genomes downloaded from GTDB. Branch with node represents bootstrap support value > 90%.


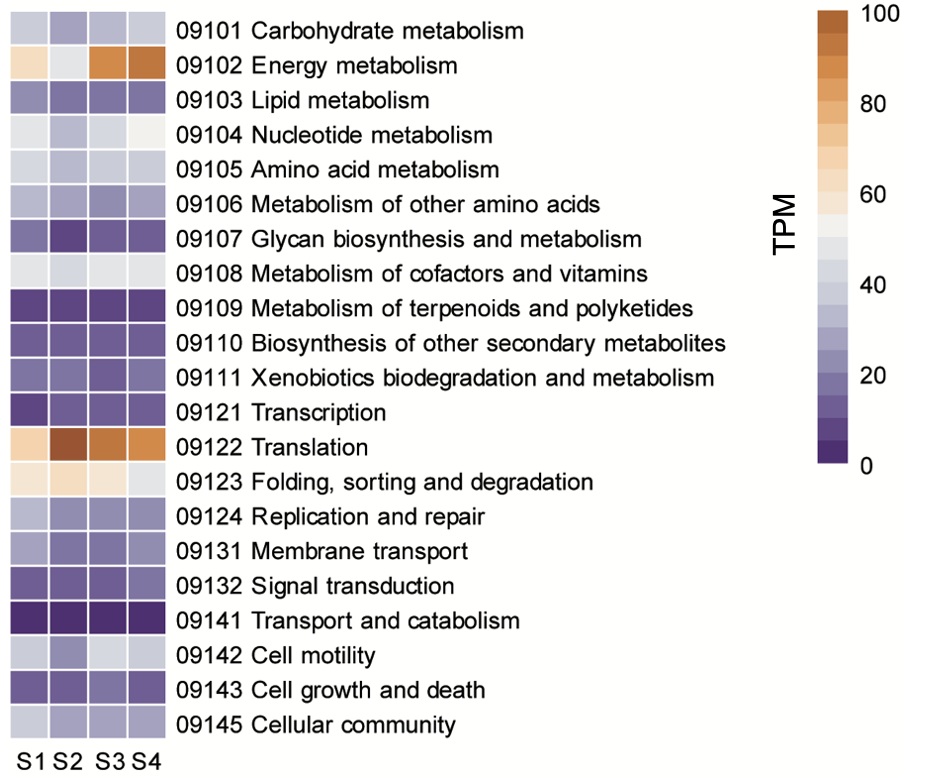


**Fig. S4** Community-level based metabolism pathway expression at different time points. S1-S4 represent the samples collected on Day 680, Day 693, Day 702, and Day 824, respectively. S2 indicates the time point when the influent was suspended.


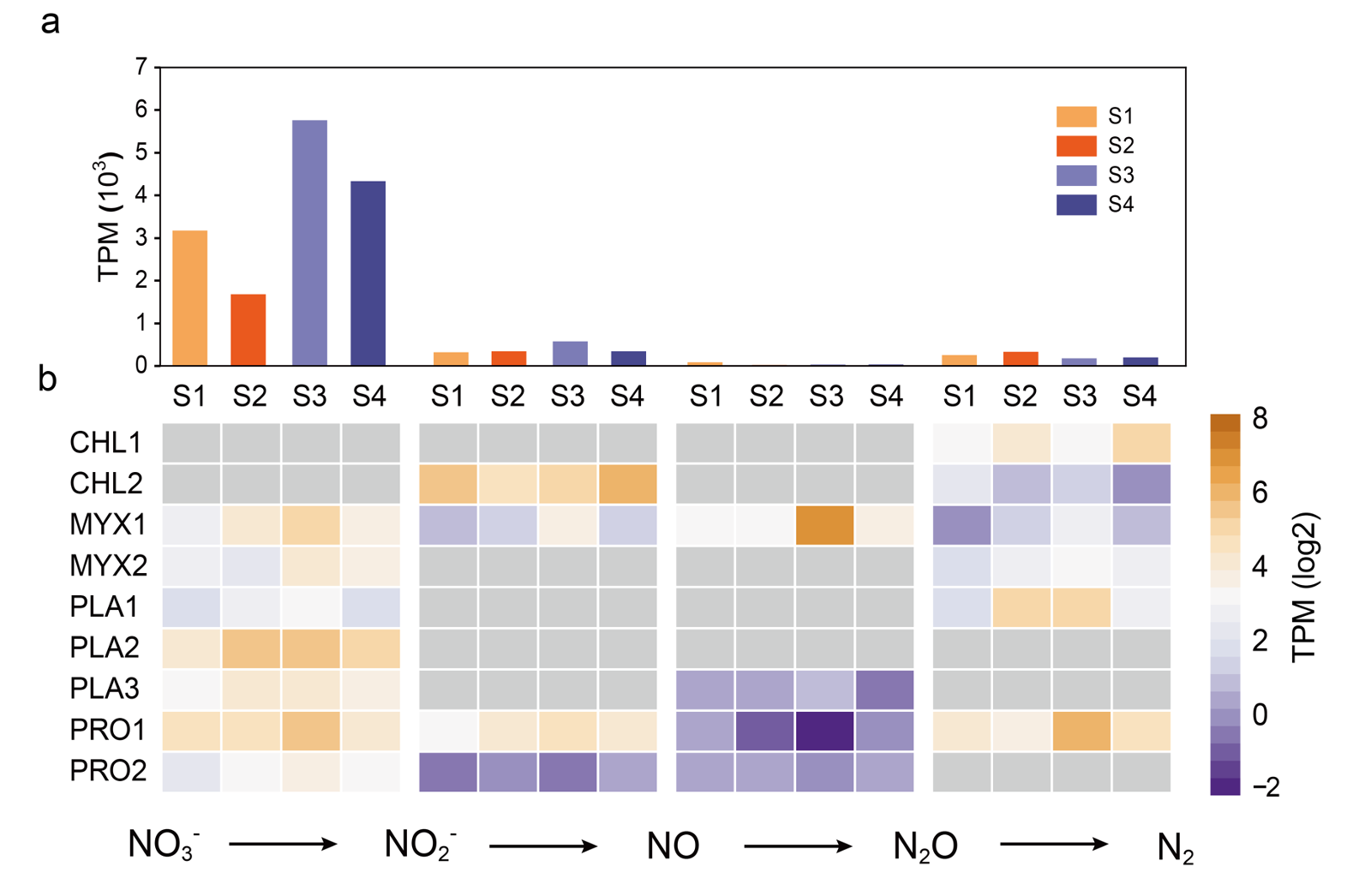


**Fig. S5** Genes involved in denitrification process. a, The expression of denitrification genes across the whole community at different time points. b, Presence and relative expression (log2 TPM) of denitrification genes across the top 9 recovered heterotrophic genomes. Gray box indicates gene absence. S1-S4 represent the samples collected on Day 680, Day 693, Day 702, and Day 824, respectively. S2 indicates the time point when the influent was suspended.


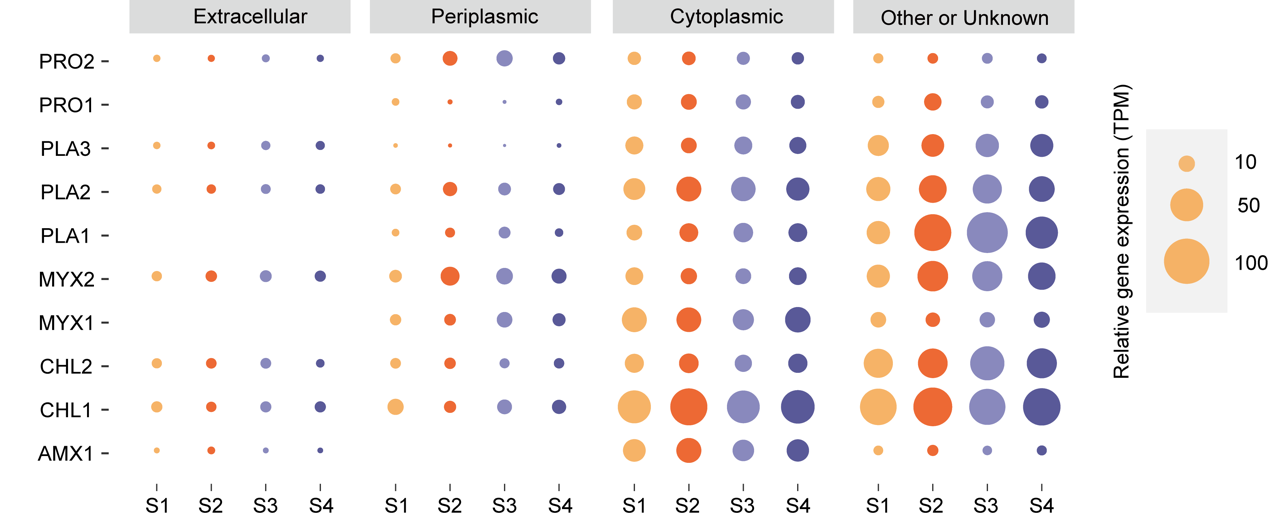


**Fig. S6** The relative gene expression of glycoside hydrolases across the recovered MAGs at different time points. The glycoside hydrolases in these recovered MAGs were identified by using HMMER [3] searching against the dbCAN database (V9) [4], and the locations of glycoside hydrolases were predicted by PSORTb [5].

**References**

1. Parks DH, Chuvochina M, Waite DW, Rinke C, Skarshewski A, Chaumeil P-a, et al. A standardized bacterial taxonomy based on genome phylogeny substantially revises the tree of life. Nat Biotechnol. 2018;36:996-1004.

2. Letunic I, Bork P. Interactive Tree Of Life (iTOL) v5: an online tool for phylogenetic tree display and annotation. Nucleic Acids Res. 2021;49:W293-6.

3. Finn RD, Clements J, Eddy SR. HMMER web server: interactive sequence similarity searching. Nucleic Acids Res. 2011;39:W29-37.

4. Zhang H, Yohe T, Huang L, Entwistle S, Wu P, Yang Z, et al. dbCAN2: a meta server for automated carbohydrate-active enzyme annotation. Nucleic Acids Res. 2018;46:W95-101.

5. Yu NY, Wagner JR, Laird MR, Melli G, Rey S, Lo R, et al. PSORTb 3.0: improved protein subcellular localization prediction with refined localization subcategories and predictive capabilities for all prokaryotes. Bioinformatics. 2010;26:1608-15.
